# TRIAD3/RNF216 E3 ligase specifically synthesises K63-linked ubiquitin chains and is inactivated by mutations associated with Gordon Holmes syndrome

**DOI:** 10.1038/s41420-019-0158-6

**Published:** 2019-03-11

**Authors:** Lukas Schwintzer, Eva Aguado Roca, Meike Broemer

**Affiliations:** Ubiquitin Signaling Group, German Center for Neurodegenerative Diseases (DZNE), Sigmund-Freud-Str. 27, 53127 Bonn, Germany

## Abstract

TRIAD3/RNF216 is a ubiquitin ligase of the RING-in-between-RING family. Recent publications identified TRIAD3 mutations in patients with neurological diseases, including Gordon Holmes syndrome and Huntington-like disorder. To understand the functional relevance of these disease-associated mutations, we have tested the ubiquitin ligase activity of mutated TRIAD3 in vitro. Several of these point mutations completely abrogated TRIAD3’s catalytic activity. Using mass spectrometry, we identified new TRIAD3-interacting proteins/substrates from mouse brain lysate, which provide a new link between TRIAD3 and processes involving clathrin-mediated endocytosis. Strikingly, we found that TRIAD3 synthesises specifically lysine-63 (K63)-linked poly-ubiquitin chains in vitro, a chain type that usually plays a role in mediating signalling events rather than triggering proteasomal degradation. Therefore, this finding is of great importance to further understand TRIAD3’s cellular role and loss-of-function in disease.

## Introduction

The E3 ubiquitin-protein ligase TRIAD3/RNF216, in the following TRIAD3, belongs to the family of RING-in-between-RING (RBR) E3 ligases. The most abundant isoform of the protein is denoted TRIAD3A, while TRIAD3B signifies the longest isoform^1^. TRIAD3A lacks amino acids 68–124 compared to TRIAD3B. Several interaction partners and potential substrates of TRIAD3 have been identified, including Toll-like receptors^1^, Tumour Necrosis factor-receptor associated factor 3 (TRAF3)^2^, the autophagy regulator Beclin^3^ and the synaptic regulator activity regulated cytoskeleton associated protein (ARC)^4^, and TRIAD3 regulated expression levels of these proteins in a ubiquitin-dependent manner.

Interestingly, two recent studies identified mutations in TRIAD3 in patients suffering from neurological/neurodegenerative disorders, indicating a critical role of TRIAD3 especially in the nervous system. Missense or point mutations were found in familial forms of Gordon Holmes syndrome, which is characterised by ataxia and fertility defects (hypogonadotropic hypogonadism)^5^. Additionally, these patients also displayed signs of dementia. A follow-up study showed that at least some of the symptoms were caused by impairment of ARC regulation due to TRIAD3 mutations^6^. Huntington-like disorder describes a disease with neurological and behavioural changes similar to Huntington’s disease but without hereditary CAG-repeat expansions in the huntingtin gene. Whole exome sequencing identified TRIAD3 mutations associated with this disease in a Belgian family^7^.

Posttranslational modifications of proteins with poly-ubiquitin chains are relevant in most cellular processes. Seven different types of poly-ubiquitin chains can be formed by conjugating ubiquitin to one of the seven lysine (K) residues of another ubiquitin molecule, giving rise to K6-, K11-, K27-, K29-, K33-, K48- or K63-linked poly-ubiquitin chains^8^. Additionally, ubiquitin chains can also be linked through ubiquitin’s first methionine (M1-/linear chains). Proteins with ubiquitin binding domains are able to bind to specific linkage types and in this way can promote downstream effects of ubiquitylation, for example by targeting proteins for proteasomal degradation or steering signal transduction pathways.

Ubiquitin E3 ligases mediate ubiquitin transfer from a conjugating enzyme (E2) to the substrate protein. Based on domain architecture and mechanism of ubiquitin transfer, E3 ligases fall in several classes. A large group of E3 ligases facilitates ubiquitin transfer via really interesting new gene (RING) domains, which are specialised cysteine- and histidine-rich Zn^2+^-coordinating motifs^9^. Ubiquitin-charged E2 enzymes bind to the RING domain and ubiquitin is transferred directly to a lysine residue of the substrate. In contrast, E3 ligases of the homologous to E6-AP C-terminus (HECT) form a thioester intermediate with ubiquitin before conjugating it to the substrate. TRIAD3 carries two RING domains and belongs to the family of RBR ligases^10^. RBR ligases function like a RING-HECT-hybrid, using their RING1 in a RING-E3-ligase manner to transfer ubiquitin to a thioester bond with a cysteine of RING2 before passing ubiquitin on to the substrate protein^11^. The human genome encodes for 14 RBR ligases^12^. Parkin and HOIL-1 interacting protein (HOIP) are the most prominent members of this family, Parkin for example is well-studied due to its role in familial Parkinson’s disease^13^ and for its mode of autoinhibition and activation^14–18^. HOIP is a particularly interesting protein because of its unique ability to ligate poly-ubiquitin chains of one specific linkage type, M1-linked chains^19,20^. Apart from HOIP, a clear linkage specificity, determined by the E3 ligase, has—to our knowledge—not been reported for other RBR ligases.

To better understand the role of TRIAD3 in human pathophysiology we investigated how disease-associated mutations affected TRIAD3’s activity as ubiquitin ligase. We found that mutations from patients with Gordon Holmes syndrome fully abrogated TRIAD3’s E3 ligase activity in vitro. Surprisingly, we also discovered that TRIAD3 preferentially ligated K63-linked ubiquitin chains in vitro and also attached K63-linked chains to substrate proteins in cells. Furthermore, we identified in an unbiased approach new interactors and substrates of TRIAD3 from mouse brain lysate, opening up new insight into TRIAD3’s biological function.

## Results

### Disease-associated TRIAD3 mutants abrogate ubiquitin E3 ligase activity

**Fig. 1:**
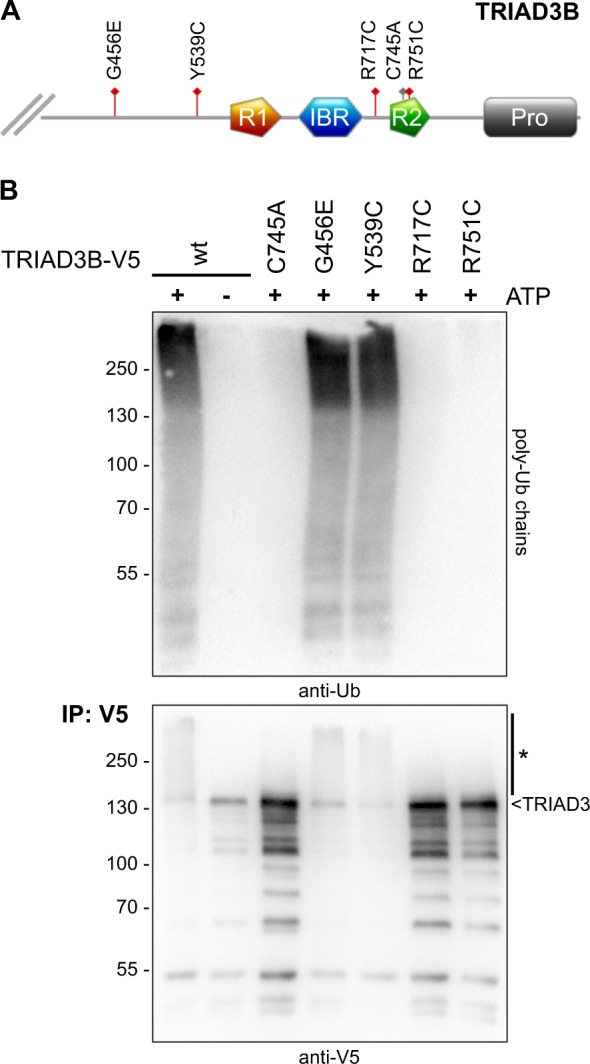
Disease mutations within the RBR domain inactivate E3 ligase activity of TRIAD3 in vitro. **a** Schematic representation of the C-terminal part of TRIAD3B showing RING1 (R1), In-between-RING (IBR), and RING2 (R2) domain (RBR domain) and proline-rich region (Pro). Disease mutations are depicted in red. Mutation of the ubiquitin acceptor site in RING2 is depicted in grey (C745A). **b** Overexpressed wt and mutant V5-tagged TRIAD3B proteins were immunopurified from HEK293T cell lysates and incubated in in vitro-ubiquitylation reactions. Free ubiquitin chains were detected by immunoblotting with anti-ubiquitin antibody (top panel). Immunopurified TRIAD3B was detected by western blotting using anti-V5 antibody (*, auto-ubiquitylated TRIAD3B)

TRIAD3 is a ubiquitin E3 ligase belonging to the RBR family of E3 ligases. Two recent publications provided a strong link between TRIAD3 and neurological diseases. Specifically, TRIAD3 mutations were identified in patients suffering from Gordon Holmes syndrome with hypogonadotropic hypogonadism, ataxia and dementia^5^. Moreover, TRIAD3 mutations were detected in patients with Huntington-like disorder (HDL)^7^. Amino acid substitutions R717C (heterozygous mutation) and R751C (homozygous) of TRIAD3B were found in patients with Gordon Holmes syndrome^5^ and lie within the catalytically active RBR domain. G456E (homozygous) and Y539C (compound heterozygous with E302*, causing premature stop), outside the RBR region, were found in HDL patients^7^ (Fig. 1a). A systematic analysis of TRIAD3’s properties as ubiquitin ligase and how these TRIAD3 mutations affect TRIAD3’s catalytic activity is currently missing. Therefore, we set off to assess whether these four disease-associated point mutations altered TRIAD3’s E3 ligase function. We expressed wild type (wt) or mutated V5-tagged-TRIAD3 constructs in HEK293T cells, immunopurified TRIAD3 via its V5-tag and tested for ubiquitin ligase activity in vitro. Wt TRIAD3 efficiently synthesised free ubiquitin chains of high molecular weight. So did also two of the point mutants, G456E and Y539C (Fig. 1b, top panel). In contrast, two point mutations found in patients with Gordon Holmes syndrome that target the RING2 domain (R751C) or a region very close to the RING2 (R717C) completely abrogated TRIAD3’s catalytic activity (Fig. 1b), comparable to the direct mutation of the catalytic cysteine in RING2, C745A (not disease-related). Of note, these mutants where also not able to auto-ubiquitylate (Fig. 1b, anti-V5 immunoblot). Our data suggest that loss of TRIAD3’s ligase function through mutations around the RBR domain can lead to neurological disease, in this case Gordon Holmes syndrome.

### Identification of TRIAD3 interaction partners from mouse cortex lysate

**Fig. 2:**
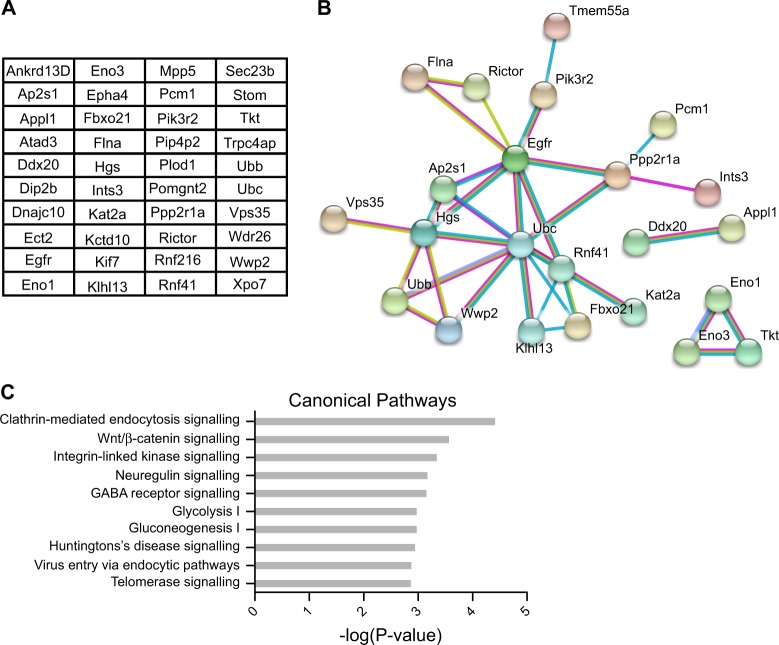
Identification of new TRIAD3 interacting proteins by mass spectrometry. **a** List of potential new TRIAD3 interactors from mouse cortex lysate. HA-TRIAD3B was affinity purified from HEK293T lysate and incubated with mouse cortex lysate. Interacting proteins were identified by mass spectrometry. Only proteins that were identified in 3 out of 3 experiments with more than 10-fold enrichment over controls were considered. **b** STRING analysis shows network between identified proteins. The type of evidence is illustrated by edge colour: blue—from curated database, magenta—experimentally determined, green—textmining. Disconnected nodes were removed. Analysis was performed with medium confidence setting (0.400). **c** Ingenuity Pathway Analysis (IPA) predicts canonical pathways for the putative interactors shown in **a**. The 10 most significantly enriched pathways are shown, pathways specific to individual cancer cell types and cytotoxic T-lymphocytes were removed

To obtain further insights into the biological function of TRIAD3, we performed pull-down experiments from mouse brain lysate with the aim to identify potential TRIAD3 interactors. In a first step, HEK293T cells were transfected with HA-tagged TRIAD3B or a HA-control plasmid and proteins were affinity purified from the lysate with anti-HA magnetic beads. Beads were then incubated with lysate from mouse cortex, washed extensively and interacting proteins were analysed by mass spectrometry. Our analysis identified a total of 40 potential interactors that were found in all three independent experiments with an enrichment of at least 10-fold compared to control samples (Fig. 2a). These mouse candidate proteins included for example ubiquitin (Ubb, Ubc) and two ubiquitin E3 ligases (RNF41, also known as E3 ubiquitin-protein ligase NRDP1 and the NEDD4-like ubiquitin E3 ligase WWP2). An analysis using the STRING database^21^ revealed connections between more than half of the potential interactors, for example through the ubiquitylation machinery, connecting Ubb/Ubc and E3 ligases as well as known ubiquitylation substrates (Fig. 2b). Further links are shown between proteins involved in endocytic processes and the epidermal growth factor receptor (EGFR).

Evaluation of the candidate list using the Ingenious Pathway Analysis (IPA®) tool predicted several canonical pathways for the putative TRIAD3 interacting proteins. Interestingly, the strongest connection was found with clathrin-mediated endocytosis signalling, e.g. through the sigma subunit of the AP2 complex (Fig. 2c).

**Fig. 3:**
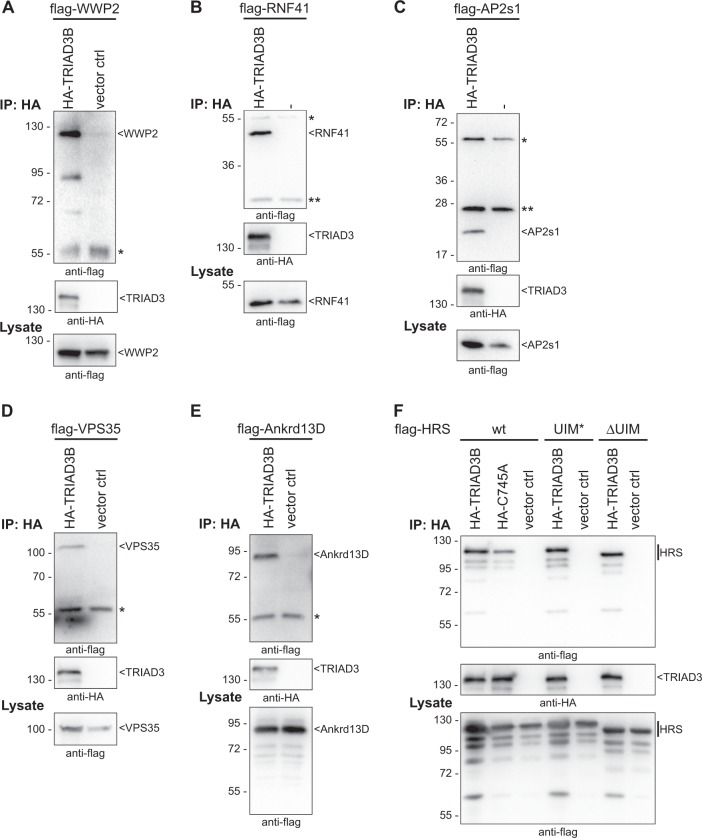
Co-immunoprecipitation of new binding partners with TRIAD3. **a**–**e** Flag-tagged candidate interactors WWP2 (**a**), RNF41 (**b**), AP2s1 (**c**), VPS35 (**d**), Ankrd13D (**e**) were co-overexpressed with HA-tagged TRIAD3B in HEK293T cells. HA-TRIAD3B was immunopurified and precipitates were analysed by western blotting using anti-flag antibody for detecting co-purified proteins (top panel) and anti-HA antibody for detecting immunopurified TRIAD3B (middle panel). Anti-flag lysate blots to illustrate expression levels of interactors are shown in the bottom panel (*, heavy and **, light chains of IP antibodies). **f** HRS interacts with TRIAD3 independent of ubiquitin chains. Immunoprecipitations were carried out as above. Additionally, catalytically inactive HA-TRIAD3B C745A was tested and co-precipitated flag-HRS similarly to the wt protein. Double point mutant UIM* (L269A, S270A) and UIM deletions mutant (ΔUIM) of HRS, which are defective in ubiquitin binding, also interacted with HA-TRIAD3B

We selected six proteins to confirm binding to TRIAD3 in co-immunoprecipitation experiments: the ubiquitin E3 ligases WWP2 and RNF41, the Adaptor Protein complex subunit sigma (AP2s1), the Vacuolar Protein Sorting-associated Protein 35 (VPS35), Hepatocyte growth factor-regulated tyrosine kinase substrate (HGS/HRS, in the following HRS) and the Ankyrin repeat domain-containing protein 13D (Ankrd13D). We selected AP2s1, VPS35, HRS and Ankrd13D, because they were all connected to endocytic or intracellular trafficking pathways^22–26^, suggesting a functional connection of TRIAD3 with these processes. HA-tagged TRIAD3B was co-expressed with flag-tagged candidate proteins in HEK293T cells and anti-HA immunoprecipitations were performed. We were able to confirm co-immunoprecipitation of all six tested proteins (Fig. 3a–f).

Two of these newly identified interactors were especially interesting in connection with the ubiquitin ligase TRIAD3: HRS carries one Ubiquitin Interacting Motif (UIM) and Ankrd13D carries even four of these ubiquitin binding domains, which have been well characterised for their ability to bind ubiquitin^27,28^. In particular, Ankrd13D and HRS bind to K63-linked ubiquitin chains and are both involved in endocytic processes^25,26,29^. We tested whether the interaction between TRIAD3 and HRS was mediated through ubiquitin chains, as for example TRIAD3 might be auto-ubiquitylated. A catalytically inactive mutant (C745A), which cannot auto-ubiquitylate, interacted with HRS similar to the wt TRIAD3 protein (Fig. 3f). Furthermore, we abrogated ubiquitin binding of HRS by either deleting the UIM of HRS (ΔUIM) or introduction of point mutations within the UIM (UIM*)^26^. Both proteins still interacted with TRIAD3 (Fig. 3f), demonstrating that HRS and TRIAD3 bind to each other independent of ubiquitin chains. We were not able to perform similar experiments for Ankrd13D, because the deletion of its UIM domains strongly destabilised the protein.

### TRIAD3 ubiquitylates AP2s1, VPS35 and Ankrd13D

Interactors of ubiquitin ligases might also be substrate proteins and get ubiquitylated by these E3 ligase. To test this, we co-expressed TRIAD3B with AP2s1, VPS35, Ankrd13D or HRS, together with His-ubiquitin, in HEK293T cells. Catalytic inactive TRIAD3B C745A served as negative control. Pulling down His-ubiquitin-conjugated proteins under denaturing conditions with Ni^2+^-NTA-agarose, we found that AP2s1, VPS35 and Ankrd13D were poly-ubiquitylated in a TRIAD3B-dependent manner (Suppl. Figure 1a-c). A catalytically inactive TRIAD3B (C745A) did not induce ubiquitylation of these proteins, confirming that the observed ubiquitin conjugation was due to TRIAD3B’s catalytic activity. In contrast, we also observed ubiquitylation of HRS in this assay but this ubiquitylation event was not abolished by the C745A mutation, suggesting in this case an indirect effect (Suppl. Figure 1d).

### TRIAD3 selectively ligates K63-linked free ubiquitin chains in vitro

To further understand the molecular and cellular function of TRIAD3, we tested whether TRIAD3 synthesised poly-ubiquitin chains of a specific linkage type. This is important as different chain types may lead to different consequences for the ubiquitylated protein.

To characterise TRIAD3’s ubiquitin ligase activity, we initially focused on a recombinant, GST-tagged TRIAD3 fragment encompassing the RBR-region. Typically, this region alone is sufficient for catalytic activity of RBR E3 ligases in vitro^30^. However, we noted that when our recombinant RBR fragment only encompassed the RBR domain and a very short stretch of additional sequence (TRIAD-RBR^825^), it was not active in an in vitro-ubiquitylation assay. A fragment including the whole C-terminal sequence following the RBR was fully active and synthesised free ubiquitin chains (Suppl. Figure 2). The RBR fragment became active when we extended it by 10 amino acids (TRIAD3-RBR^835^), indicating that further residues than just the RBR region itself are required for activity. For further experiments the equally active construct TRIAD3-RBR^845^ was used.

**Fig. 4:**
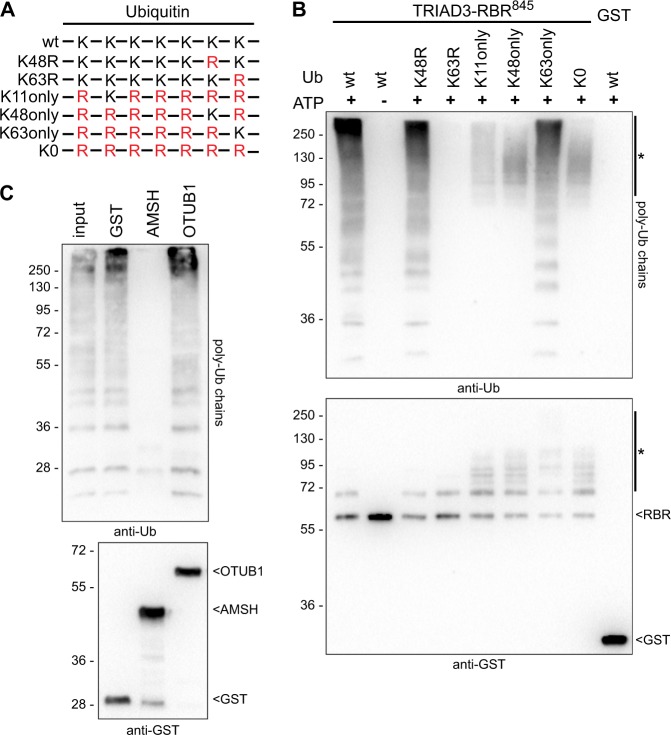
TRIAD3 selectively ligates unanchored, K63-linked ubiquitin chains in vitro. **a** Scheme of ubiquitin mutants used in (4b), illustrating ubiquitin’s seven internal lysines (K) and their replacement by arginine (R), shown in red. **b** GST-fused TRIAD3-RBR^845^ fragment was incubated with wt ubiquitin or different ubiquitin mutants as illustrated in **a**. As negative controls, reactions were incubated without ATP or with GST instead of GST-TRIAD3-RBR^845^. Samples were analysed by western blotting and ubiquitin chains were detected using anti-ubiquitin antibodies (upper panel), and GST-TRIAD3-RBR fragments were detected using anti-GST antibodies (lower panel). * denotes multi-mono-ubiquitylated GST-TRIAD3-RBR in K11, K48, and K0 ubiquitin samples. **c** Ubiquitin chains synthesised by recombinant TRIAD3A were analysed by UbiCRest with K48-specific (OTUB1) or K63-specific (AMSH) de-ubiquitylating enzymes or GST as control. Ubiquitin chains and GST and GST-fused de-ubiquitylating enzymes were detected by immunoblotting with anti-ubiquitin (upper panel) and anti-GST antibodies (lower panel), respectively

We then addressed the question whether TRIAD3 ligated ubiquitin chains of a certain linkage type. For this, we used either wt ubiquitin or several ubiquitin mutants in which one or several of the seven internal lysines in the ubiquitin sequence had been replaced by arginine (Fig. 4a). Strikingly, TRIAD3-RBR^845^ ligated free ubiquitin chains with wt ubiquitin and equally well with K48R-mutated ubiquitin but was completely inactive with K63R-mutated ubiquitin (Fig. 4b). This suggests that TRIAD3 requires K63 of ubiquitin to produce poly-ubiquitin chains. In line with these results, TRIAD3 was able to catalyse chain formation with a ubiquitin mutant, which contains only K63 but no other lysine residues. In contrast, when incubated with K11-only, K48-only or lysine-less (K0) ubiquitin, TRIAD3 did not ligate ubiquitin chains but instead seemed to auto-ubiquitylate with multiple mono-ubiquitin units (Fig. 4b, lower panel and upper part of the anti-ubiquitin blot). Under conditions where TRIAD3 ligated free ubiquitin chains, only moderate mono-ubiquitylation with one ubiquitin unit was observed, indicating that TRIAD3 preferentially ligates K63-linked chains rather than auto-ubiquitylating itself. In accordance with previously published results, TRIAD3 was active with the ubiquitin conjugating enzymes (E2) UbcH5 and UbcH7^4^ and K63-linked chains were ligated with both E2s (Figs. 1b, 2b: UbcH5b; UbcH7: data not shown).

Ubiquitin chain restriction analysis (UbiCRest) uses chain type-specific de-ubiquitylating enzymes (DUBs) to determine linkage types of ubiquitin chains in vitro^31^. To further validate the linkage type of ubiquitin chains ligated by TRIAD3, recombinant full length TRIAD3 was used to synthesise ubiquitin chains in vitro (Fig. 4c, input lane). These chains were either incubated with GST as control or with two chain-type specific DUBs: Ubiquitin thioesterase OTUB1 specifically cleaves K48-linked chains while STAM binding protein/AMSH exclusively cuts K63-linked chains. Immunoblot analysis revealed that ubiquitin chains were completely degraded by AMSH but neither by OTUB1 nor the GST control (Fig. 4c), further supporting our finding that TRIAD3 synthesises K63-linked ubiquitin chains in vitro, full length TRIAD3 protein as well as the TRIAD3-RBR^845^ fragment.

### TRIAD3 attaches K63-linked ubiquitin chains to substrate proteins in cells

**Fig. 5:**
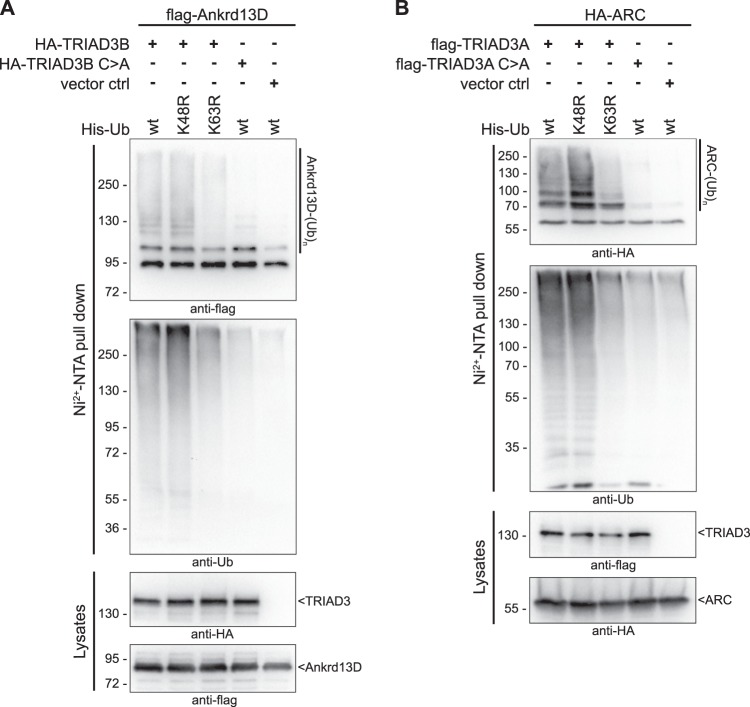
TRIAD3 attaches K63-linked ubiquitin chains to ARC and Ankrd13D. **a** HA-tagged TRIAD3B and flag-Ankrd13D were co-overexpressed with His-tagged wt ubiquitin or His-tagged K48R or K63R mutant ubiquitin. Catalytic inactive HA-TRIAD3B C745A or HA peptide were co-expressed with flag-Ankrd13D and His-ubiquitin as controls. His-ubiquitylated proteins were precipitated with Ni^2+^-NTA affinity beads from HEK293T lysates under denaturing conditions, separated by SDS-PAGE and analysed by immunoblotting with anti-flag or anti-ubiquitin antibody. Protein expression was analysed in lysates by anti-HA and anti-flag immunoblot. **b** Ubiquitylation of ARC by TRIAD3A was analysed as in **a**

After observing the exclusive formation of K63-linked ubiquitin chains in vitro, we asked whether TRIAD3 also attached such poly-ubiquitin chains to substrate proteins. To test this, we chose our newly identified substrate Ankrd13D and the previously described ARC. ARC is involved in the regulation of synaptic plasticity^32–34^. TRIAD3A has been shown to finetune ARC levels by ubiquitylation and proteasomal degradation^4,6^. To test which chain type was conjugated by TRIAD3 to these substrate proteins, we co-expressed flag-Ankrd13D with HA-TRIAD3 and His-ubiquitin or HA-ARC together with flag-TRIAD3 and His-ubiquitin as described above. Flag-Ankrd13D and HA-ARC were poly-ubiquitylated in a TRIAD3-dependent manner and a catalytically inactive TRIAD3 (C>A) did not enhance ubiquitylation (Fig. 5). To test the requirement of lysine-48 vs. lysine-63 of ubiquitin for TRIAD3-dependent ARC ubiquitylation, we used His-tagged K48R-ubiquitin or His-K63R-ubiquitin mutants. Ubiquitylation was strongly impaired for both substrate proteins with the K63R mutant ubiquitin, suggesting that also under these conditions, TRIAD3 produced preferentially K63-linked chains. In contrast, replacing K48 with arginine (K48R) did not reduce ubiquitylation. Comparing ARC and Ankrd13D levels in the presence or absence of active TRIAD3, we did not notice any loss or degradation after co-expression of TRIAD3 (Fig. 5, lysate panels), supporting our finding that TRIAD3 ligated K63-linked ubiquitin chains that do not target proteins for proteasomal degradation.

Together, we show that mutations of TRIAD3, found in Gordon Holmes syndrome with hypogonadism, ataxia and dementia, directly abrogate its catalytic activity. We identified proteins that interact with TRIAD3 and get ubiquitylated in a TRIAD3-dependent manner. Furthermore, we provide striking evidence that TRIAD3 has a clear specificity for synthesising K63-linked ubiquitin chains. These findings ask for future research to address how its K63-specific activity regulates important cellular processes and prevents disease.

## Discussion

We have addressed the question how disease-associated mutations of the E3 ligase TRIAD3 affect its catalytic function. Our results provide in vitro evidence that point mutations found in patients with Gordon Holmes syndrome^5^ abrogate TRIAD3’s E3 activity. Of note, further missense or frame shift mutations that were identified in the same study lead to truncated TRIAD3 protein variants, lacking the catalytic RBR part, and therefore also lack E3 activity, supporting our notion that the catalytic activity is fundamental for protein function. In contrast, it remains to be elucidated how TRIAD3 mutations from HDL patients^7^ that lie outside the RBR domain and did not abrogate E3 activity, affect TRIAD3 function and lead to disease. Possibly, these point mutations might disrupt specific protein-protein interactions.

Our study characterised the catalytic activity and specificity of the RBR E3 ligase TRIAD3. We observed a clear specificity for generation of K63-linked poly-ubiquitin chains in vitro. To our knowledge, specific synthesis of only one type of ubiquitin chain, determined by the E3 ligase itself and not the E2 enzyme, has so far only been demonstrated for one other RBR ligase. HOIP synthesises M1-linked ubiquitin chains exclusively^19^, and this specificity is determined by a region following the RBR, named Linear ubiquitin chain Determining Domain (LDD)^20^. It will be exciting to elucidate the structural basis for the K63-linkage specificity, similarly as it has been done for HOIP^35,36^. Furthermore, follow-up studies will be necessary to confirm that TRIAD3 forms K63-linked chains also in vivo and if so, under which physiological conditions.

We have found that the RBR domain of TRIAD3 alone is not sufficient for ubiquitin ligase activity in vitro but requires an additional C-terminal extension. Interestingly, a recent article has reported that the region C-terminal to the RING2 domain of TRIAD3 is rich in cysteine and histidine residues and coordinates a third Zn^2+^-ion in addition to two Zn^2+^-ions that are associated with the RING2 domain^30^. It is possible that our observation of around 50 aa following the RING2 being necessary for activity can be explained by the requirement for an additional Zn^2+^-binding site, residing in this region. While Martino et al. only observed modest catalytic activity of TRIAD3-RBR, they noted weak formation of K63-linked di-ubiquitin, which is in line with the chain type specificity we describe here.

As TRIAD3 synthesised free K63-linked chains in vitro, an interesting question is whether this is also the case with substrate proteins. We observed that TRIAD3-dependent ubiquitylation of ARC and Ankrd13D was abrogated when lysine-63 of ubiquitin was replaced by arginine, indicating that also in this overexpression setting, TRIAD3 conjugated preferentially K63-linked poly-ubiquitin chains onto a substrate. We have not been able to achieve ARC or Ankrd13D ubiquitylation by TRIAD3 in a pure in vitro experiment. One possible reason for this might be TRIAD3’s property to synthesise specifically chains of one linkage type, and therefore it might not be able to directly ubiquitylate a substrate. A priming mono-ubiquitylation step, performed by another E3 ligase might be required and would be missing in an in vitro set-up. Candidate E3s for this task are RNF41 and WWP2, which we have identified as new TRIAD3 interactors. Mono-ubiquitylation in some cases can be mimicked by N- or C-terminal fusion of ubiquitin to the substrate protein. However, this has also not enabled in vitro-ubiquitylation in our hands, leaving the question unanswered why TRIAD3 does not ubiquitylate substrates in vitro and whether the tested proteins are bona fide substrate proteins.

Two previous studies have described ARC as a substrate of TRIAD3 and showed ARC degradation in a TRIAD3-dependent manner^4,6^. In contrast, we have not observed ARC degradation upon TRIAD3 overexpression. Our data point to a non-degradative mode of protein ubiquitylation by TRIAD3, according to a well-studied role of K63-linked poly-ubiquitylation in mediating signalling events, for example by providing interaction platforms for proteins with specific ubiquitin binding domains^37^. Our experiments also indicated that only a small fraction of ARC protein is ubiquitylated under basal conditions. This has hampered so far a more thorough analysis of ubiquitylated cellular ARC, e.g. with an UbiCRest assay. Thus, ARC might only be a substrate for TRIAD3 under specific physiological conditions, e.g. in fine-tuning of synaptic plasticity^4^. Interestingly, in this setting, ARC is involved in regulation of postsynaptic endocytosis of AMPA receptors in a clathrin-dependent manner. In line with this, several of our newly identified interactors are also relevant for clathrin-mediated endocytosis, supporting a role of TRIAD3 in this context.

In conclusion, our study describes an unanticipated chain type specificity of the RBR ligase TRIAD3 and inactivation of this E3 ligase activity by disease-associated mutations. Together with our newly identified interacting proteins and pathways, these findings open up new perspectives and potential lines of research. Future work will have to address the relevance of K63-ubiquitylation by TRIAD3 in vivo and improve understanding why loss of TRIAD3 function leads to disease.

## Materials and methods

### Chemicals and antibodies

Chemicals and reagents were purchased from Carl Roth or Sigma-Aldrich. Cell culture reagents were obtained from Gibco™ (Thermo Fisher Scientific) or PAN Biotech, unless stated otherwise.

Anti-ubiquitin (Millipore, 07-375; LifeSensors, VU101), anti-GST (3–4 C) (Invitrogen™/Thermo Fisher Scientific, 13-6700), anti-flag M2 (Sigma-Aldrich, F1804), anti-HA (Roche, 11867423001), and anti-V5 (BIO-RAD, MCA1360) were used as primary antibodies. Horseradish peroxidase (HRP)-coupled secondary antibodies were from Santa Cruz Biotechnology or SouthernBiotech.

### DNA constructs

Expression constructs for human TRIAD3B (NCBI accession number NM_207111.3) were cloned into a modified pcDNA3 vector coding for a C-terminal V5/His_6_-tag (kindly provided by Tencho Tenev) or pN3HA vector with 3X N-terminal HA-tag (kindly provided by Christoph Thiele). cDNA coding for TRIAD3A was cloned into pCMV-3Tag-6 (Agilent) or pGEX-6P-1 (GE Healthcare). Constructs coding for TRIAD3B C-terminus (aa 562-923) or TRIAD3B-RBR fragments (aa 562-825/-835/-845) were cloned into pGEX-6P-1 (). Catalytically inactive point mutant TRIAD3B C745A (TRIAD3A C688A) and disease point mutants TRIAD3B G456E, Y539C, R717C and R751C were produced by site-directed mutagenesis (SDM).

cDNA coding for human Ankrd13D (IMAGE ID 5210878) was cloned into pCMV-3Tag-6. psigma2-EGFP was a gift from Tom Kirchhausen (Addgene plasmid # 53610) and was used as template to clone rat AP2s1 into pCMV-3Tag-6. Human ARC cDNA (IMAGE ID 4555433) was cloned into pN3HA. pCS2 HRS-RFP was a gift from Edward De Robertis (Addgene plasmid # 29685) and expression constructs for HRS and UIM deletion mutant HRS ΔUIM (Δ aa 258–277) were cloned into pCMV-3Tag-6. Mutant UIM construct HRS UIM* (L269A, S270A) was produced by SDM. Human VPS35 cDNA (IMAGE ID 30340379) was cloned into pCMV3Tag-6. pCW7 coding for His_6_/c-myc-tagged yeast ubiquitin and ubiquitin mutant constructs K48R and K63R were a kind gift from Ron Kopito.

### Recombinant protein purification

GST-fusion proteins were purified as described previously^38^. Upon affinity purification immobilised TRIAD3-RBR fragments were eluted from Glutathione Sepharose™ 4B beads (GE Healthcare) in a buffer containing 20 mM Tris (pH 8.0), 100 mM NaCl, 40 mM glutathione, 1 mM EDTA, and 50 µM ZnCl_2_. Glutathione later was removed using Zeba™ spin desalting columns (Thermo Scientific).

Immobilised full length TRIAD3A was cleaved off from GST over night at 4 °C by PreScission™ protease (GE Healthcare) in cleavage buffer containing 50 mM Tris (pH 8.0), 250 mM NaCl, 1 mM EDTA, 0.5 % Triton X-100, and 1 mM dithiothreitol (DTT).

### In vitro-ubiquitylation reaction

In vitro-ubiquitylation samples contained 10 mM ATP, 15 mM ubiquitin (Sigma-Aldrich), 0.6 µM UbcH5b (BostonBiochem^®^), 0.1 µM E1 (BostonBiochem^®^), and 1 µM of recombinant TRIAD3 proteins or wild type and mutant TRIAD3B immobilised to anti-V5 agarose beads in a buffer of 20 mM Hepes (pH 8.0), 150 mM NaCl, 10 mM MgCl, and 0.5 mM DTT. Ubiquitylation reactions were carried out for 90 min at 37 °C and stopped for western blot analysis by the addition of Laemmli sample buffer.

### Ubiquitin chain restriction analysis (UbiCRest)

UbiCRest was carried out according to Hospenthal and colleagues^31^. Cysteine protease DUB OTUB1 (Ubiquigent™) was diluted at 5 µM in a 5X solution containing 25 mM Tris (pH 7.5), 150 mM NaCl, and 10 mM DTT and incubated for 15 min at room temperature to reach full activity. For UbiCRest ubiquitin chains from an in vitro-ubiquitylation reaction with recombinant TRIAD3A were incubated with 1 µM OTUB1, 1 µM AMSH (Ubiquigent™) or 1 µM GST as control for 30 min at 37 °C in a buffer containing 50 mM Tris (pH 7.5), 50 mM NaCl, and 5 mM DTT. Reactions were stopped by the addition of Laemmli sample buffer and analysed by immunoblotting.

### Transfection and immunoprecipitation

HEK293T cells were cultured in DMEM with 10% fetal bovine serum, 50 units/ml penicillin, and 50 µg/ml streptomycin at 37 °C and 5% CO_2_. Cells were transfected as indicated in 10-cm dishes using calcium phosphate and harvested 24–48 h after transfection.

Upon overexpression HEK293T cells were lysed in a buffer containing 125 mM Tris (pH 7.5), 150 mM NaCl, 1% Triton X-100, 10% glycerol, 1 mM EDTA, cOmplete™ protease inhibitor (Roche), and 1 mM DTT. Cell debris was spun down and supernatants were incubated for 4 h at 4 °C with anti-HA agarose affinity gel or anti-V5 agarose affinity gel (both Sigma-Aldrich). Immobilised protein was washed for 4 times in wash buffer containing 10 mM Tris (pH 7.5), 150 mM NaCl, 0.1% Triton X-100, and 5% glycerol. For interaction studies immobilised protein complexes were eluted with 100 mM glycine (pH 2.5) and analysed by immunoblotting.

### His-ubiquitin pull-down

His-pull-down of ubiquitylated proteins from HEK293T cell lysates was performed for 4 h at room temperature as described previously^38^.

### Western blot analysis

Proteins were separated by SDS-PAGE and transferred onto a polyvinylidene fluoride (PVDF) membrane (Merck) by semi-dry blotting. Membranes were blocked with 5% milk in PBS and incubated overnight at 4 °C in primary antibody dilution. Membranes were washed three times in PBS with 0.5% Tween® 20 and incubated in HRP-coupled secondary antibody dilution for 1 h at room temperature. Membranes were washed again and proteins were detected using WesternBright™ enhanced chemiluminescence (ECL) HRP substrate (advansta) and a ChemiDoc™ imaging system (BIO-RAD).

### Preparation of mouse brain lysate

All animal experiments were performed in compliance with the guidelines for the welfare of experimental animals issued by the Federal Government of Germany. The brains of 6–8-week-old C57BL/6 mice were dissected to separate the cortex, followed by lysis in ice-cold homogenising buffer (0.32 M sucrose, 50 mM EDTA, 2 mM HEPES, pH 7.4, cOmplete™ protease inhibitor (Roche)) in a Teflon-Glass homogeniser (7 strokes). Homogenates were supplemented with 2% Triton X-100, incubated at 4 °C for 2 h and cleared by ultracentrifugation with an F50L-24 × 1.5 Rotor (Thermo Fisher Scientific) at 20,000 rpm for 10 min. The protein concentration of the lysates was roughly estimated using a NanoDrop instrument (Thermo Fisher Scientific).

### HA-affinity purification

HEK293T cells were transfected with pN3HA-TRIAD3B or control vector. 48 h post transfection, cells were lysed for 1 h in ice-cold lysis buffer (20 mM Tris-HCl, pH 8, 150 mM NaCl, 1% Triton X-100, cOmplete™ protease inhibitor (Roche), 1 mM DTT, PhosSTOP phosphatase inhibitor). Lysates were incubated with HA-magnetic beads (Pierce) over night at 4 °C. Afterwards, beads were washed twice with low salt buffer (20 mM Tris-HCl, pH 8, 100 mM NaCl, 0.1% Triton X-100), twice with high salt buffer (20 mM Tris-HCl, pH 8, 150 mM NaCl, 0.1% Triton X-100), and again twice with low salt buffer. HA-TRIAD3 immobilised to beads was incubated with approx. Five milligrams of protein in mouse cortex lysate at 4 °C for 5 h. Beads were washed 4x in PBS, 0.5% Triton X-100 and subsequently incubated in Laemmli sample buffer for 5 min at 95 °C. The HA-IP eluates were separated on 4–12% Bis-Tris gradient gels (NuPAGE, Life Technologies) and stained with SimplyBlue Coomassie G-250 SafeStain (Invitrogen). Gel slices were excised for analysis by LC-MS/MS.

### Mass spectrometric analysis

#### Peptide preparation

For protein identification gel slices were subjected to in-gel digestion^39,40^. In brief, slices were washed consecutively with water, 50% acetonitrile (ACN), and 100% ACN. Proteins were reduced with 20 mM DTT in 50 mM ammonium bicarbonate and alkylated with 40 mM iodoacetamide (in 50 mM bicarbonate) in the dark for 30 min. The slices were washed again and dehydrated with ACN. Dried slices were incubated with 330 ng trypsin (sequencing grade, Promega) at 37 °C o/n. The peptide extract was separated and remaining peptides extracted with 50% ACN. Peptides were dried in a vacuum concentrator and stored at −20 °C.

#### LC-MS measurements

Peptides were dissolved in 0.1% TFA and 1/3 was injected onto a C18 trap column (20 mm length, 100 µm inner diameter, ReproSil-Pur 120 C18-AQ, 5 µm, Dr. Maisch GmbH) made in-house. Bound peptides were eluted onto a C18 analytical column (200 mm length, 75 µm inner diameter, ReproSil-Pur 120 C18-AQ, 3 µm, with 0.1% formic acid as solvent A). Peptides were separated during a linear gradient from 2 to 35% solvent B (90% acetonitrile, 0.1% FA) within 80 min at 350 nl/min. The nanoHPLC was coupled online to an LTQ Orbitrap Velos mass spectrometer (Thermo Fisher Scientific). Peptide ions between 330 and 1600 m/z were scanned in the Orbitrap detector with a resolution of 30,000 (maximum fill time 400 ms, AGC target 106). The 25 most intense precursor ions (threshold intensity 3000, isolation width 1.0 Da) were subjected to collision induced dissociation (CID, normalized energy 35) and analysed in the linear ion trap. Fragmented peptide ions were excluded from repeat analysis for 15 s.

### Data analysis

Raw data processing and analysis of database searches were performed with Proteome Discoverer software 2.1.0.81 (Thermo Fisher Scientific). Peptide identification was done with an in-house Mascot server version 2.5 (Matrix Science Ltd). MS2 data were searched against *Mus musculus* sequences in SwissProt (release 2016_02) and common contaminants. Precursor ion m/z tolerance was 8 ppm, fragment ion tolerance 0.5 Da. Tryptic peptides with up to two missed cleavages were searched. Carbamidomethylation of cysteines was set as static modification. Oxidation of methionine and N-terminal protein acetylation were allowed as dynamic modifications. Mascot results were assigned q-values by the percolator algorithm^41^ version 2.05 as implemented in Proteome Discoverer. Spectra of peptide spectrum matches (PSMs) with *q* > 0.01 were sent to a second round of database search with semitryptic enzyme specificity (one missed cleavage allowed) where carbamidomethylation was searched as a dynamic modification. Proteins were included if at least two peptides were identified with *q* ≤ 0.01. False positive rates were estimated to be 0.8, 1.2 and 1.0% on PSM, peptide, and protein level respectively.

Proteins that were identified in all three independent experiments, each with ≥10 fold over control samples (based on peak area) were considered putative interactors. Mitochondrial proteins were disregarded.

### Ingenuity pathway analysis (IPA®)

The analysis was performed with IPA software (QIAGEN) version 01–07^42^.

## Supplementary information

Suppl Figure 1

Suppl Figure 2

Supplemental Material File #1
