# Supplementary figures and images for: TRIAD3/RNF216 E3 ligase specifically synthesises K63-linked ubiquitin chains and is inactivated by mutations associated with Gordon Holmes syndrome
Source: Cell Death Discov. 2019 Mar 11;5:75. doi: 10.1038/s41420-019-0158-6 (PMC6411869; doi:10.1038/s41420-019-0158-6)

# Supplementary Figure 1

**A**

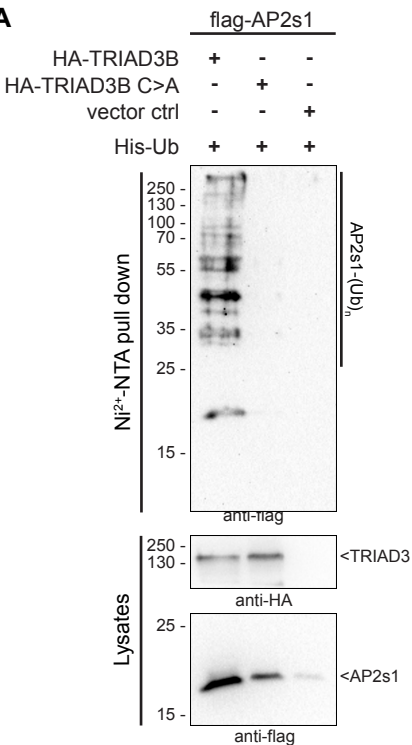

**B**

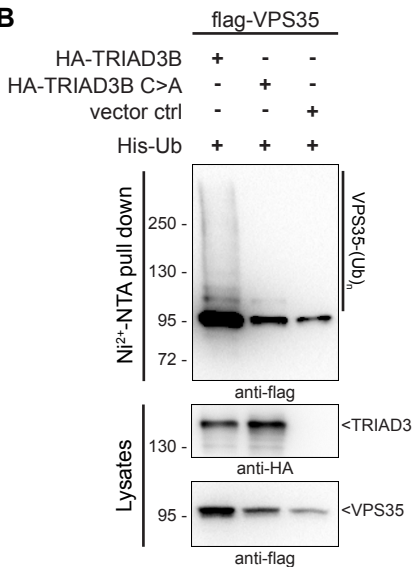

**C**

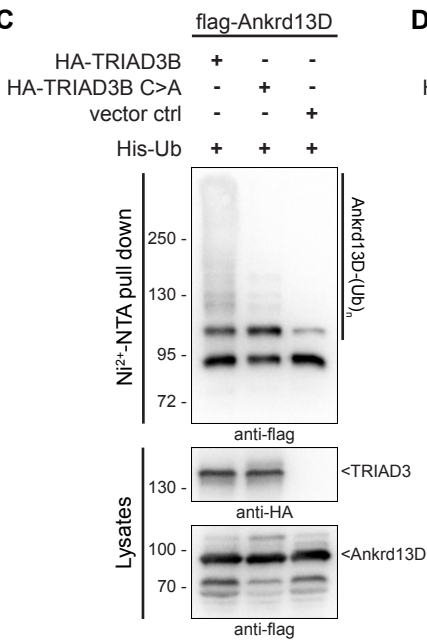

**D**

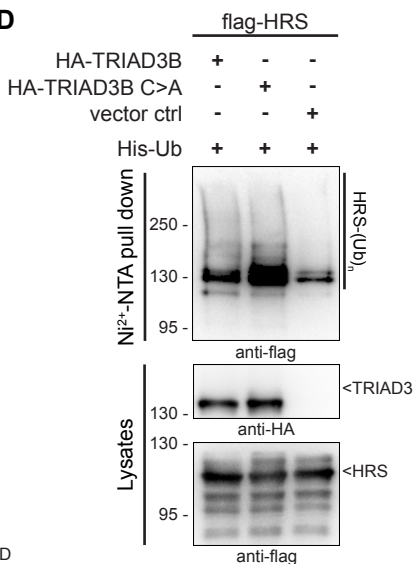

Supplement: Supplementary file 1 — Suppl Figure 1 [file 41420_2019_158_MOESM1_ESM.pdf]

# Supplementary Figure 2

**A**

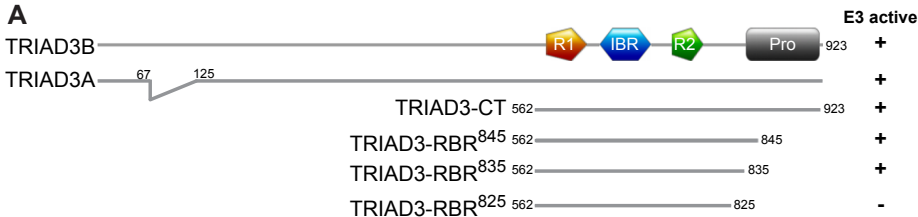

**B**

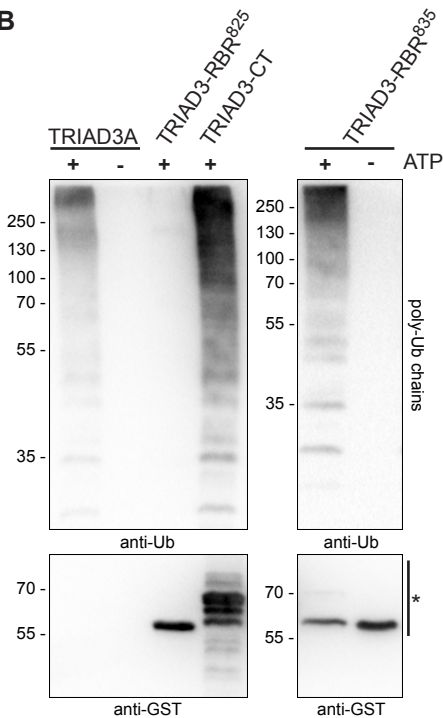

Supplement: Supplementary file 2 — Suppl Figure 2 [file 41420_2019_158_MOESM2_ESM.pdf]
