# Supplementary material for: TRIAD3/RNF216 E3 ligase specifically synthesises K63-linked ubiquitin chains and is inactivated by mutations associated with Gordon Holmes syndrome
Source: Cell Death Discov. 2019 Mar 11;5:75. doi: 10.1038/s41420-019-0158-6 (PMC6411869; doi:10.1038/s41420-019-0158-6)
Supplement: Supplementary file 3 — Supplemental Material File #1 [file 41420_2019_158_MOESM3_ESM.docx]

**Supplementary Figure 1**

A-D) Flag-tagged AP2s1 (A), VPS35 (B), Ankrd13D (C) or HRS (D) were co-overexpressed with wt HA-TRIAD3B and His-ubiquitin or with His-ubiquitin and catalytically inactive HA-TRIAD3B C745A or empty vector as controls. His-ubiquitylated proteins from HEK293T lysates were immobilised to Ni^2+^-NTA affinity beads under denaturing conditions, separated by SDS-PAGE and analysed by western blotting with anti-flag antibodies. Protein expression was analysed in HEK293T lysates by immunoblotting with anti-HA and anti-flag antibodies (“Lysate” panels).

**Supplementary Figure 2**

***In vitro*-activity of recombinant TRIAD3 fragments requires an extended RBR domain.**

A) Schematic representations of the longest splice variant TRIAD3B showing its RBR domain (R1, RING1; IBR, In-between-RING; R2, RING2) (aa 562-778 of TRIAD3B) and proline-rich region (Pro). Splice variant TRIAD3A lacks amino acids (aa) 68-124 compared to TRIAD3B. Recombinant TRIAD3B C-terminal (CT) and RBR fragments with varying length have been used in Suppl. Fig. 1B).

B) *In vitro*-ubiquitylation assay using recombinant TRIAD3A and GST-tagged TRIAD3 fragments. As negative control, reactions were incubated without ATP. Samples were analysed by western blotting and ubiquitin chains were detected using anti-ubiquitin antibodies (upper panel), GST-TRIAD3-RBR fragments were detected using anti-GST antibodies (lower panel). TRIAD3-RBR^825^ does not show E3 ligase activity. Longer fragments, minimally comprising aa 562-835, and full length TRIAD3A synthesised unanchored poly-ubiquitin chains.
